# Scleral fixation of the Smaller-Incision New Generation Implantable Miniature Telescope (SING IMT™)

**DOI:** 10.1007/s00417-024-06444-7

**Published:** 2024-03-25

**Authors:** Nicole Eter, Oliver Behr

**Affiliations:** Department of Ophthalmology, University of Muenster Medical Center, Albert-Schweitzer-Campus 1, Building D15, 48149 Muenster, Germany

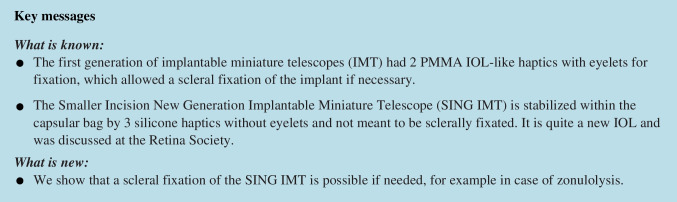



**Fig. 1:**
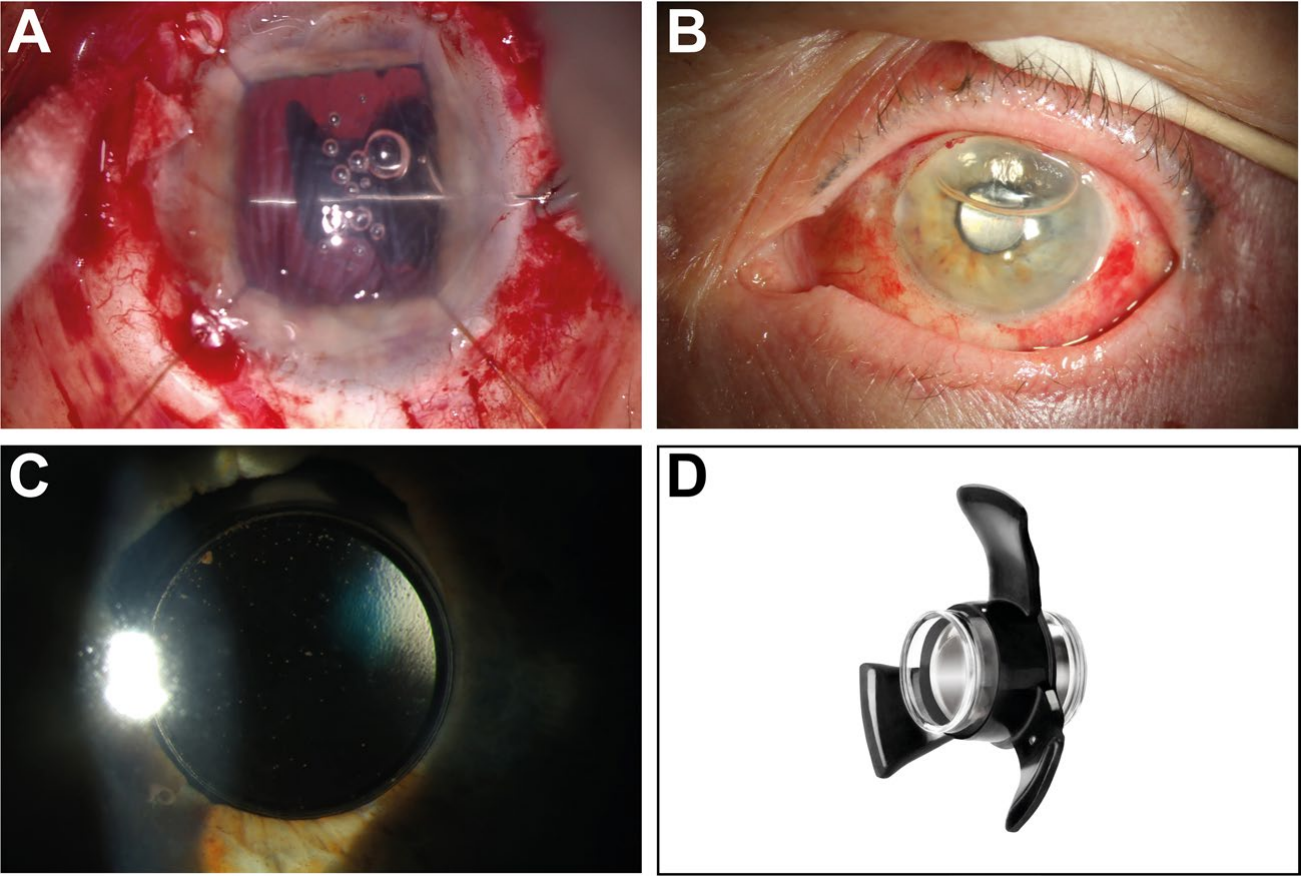
NG SI IMT 3X intraoperative and postoperative status in the first ten days. **A** Intraoperative photo showing the suturing of the haptic and a scleral flap. **B** First postoperative day, air bubble still in the anterior chamber, device centered. **C** Postoperative day 10, device perfectly centered **D** NG SI IMT 3X (curtesy of Samsara Vision Inc)

Dear Editor,

The Smaller-Incision New Generation Implantable Miniature Telescope (SING IMT™) is the second-generation Galilean telescope implant designed to improve visual acuity in patients with geographic atrophy. It received EMA approval in 2020 [1]. Images are magnified 2.7 × and projected onto healthy photoreceptors surrounding the macula, reducing the impact of the central scotoma. It consists of two basic components: a central Quartz glass optics and a silicone carrier (Fig. 1D). The device is stabilized within the capsular bag by three silicone haptics instead of two PMMA IOL-like haptics with eyelets [2]. Scleral suturing of the first generation implant has already been described in case of capsule rupture or zonular dialysis and could be done using the eyelets within the PMMA haptics [3]. The silicone haptics of the present 3X do not include eyelets and are designed for in capsular placement only [4].

A 78-year-old female patient presented to our hospital with bilateral geographic atrophy and bilateral cataract. ETDRS visual acuity was 30 letters in the right eye and seven letters in the left eye. Anterior segments were unremarkable except for a cataract on both eyes. Fundoscopy displayed bilateral geographic atrophy with an area of right 71.44 mm^2^ and left 79.82 mm^2^. A central island of functional photoreceptors was no longer present. The patient was informed about the Miniature Telescope NG SI IMT™ 3X (Samsara Vision, Inc., Far Hills, NJ, USA) and agreed to receive the implant.

During surgery two paracenteses were created at 3 and 9 o’clock as well as a 2.4-mm scleral tunnel. Cataract extraction using phaco chop technique was achieved uneventfully. The capsular bag was filled with viscoelastic, the scleral incision was increased to 7.5 mm, and the SING implant was implanted using the device-specific shooter. During implantation one of the haptics temporal inferiorly did not slide into the capsular bag. After removal of the injector, zonulolysis was seen in the area between 3 and 6 o’clock, and the haptic at 4.30 o’clock was surrounded by vitreous. Anterior vitrectomy was performed. The pupil became increasingly miotic to the point where we decided to insert five iris retractors to enhance the view. An attempt was made to maneuver the haptic into the capsular bag using a push–pull instrument, which was not successful. Instead, further shear stress on the zonula fibers was seen with further zonulolysis.

At this point, there were only two options: removal of the implant, or a scleral fixation of the haptics. The decision was made to remove the capsule and to suture the mini telescopic IOL to the sclera. Three scleral flaps were created (Fig. 1A). Using a double-armed 10–0 prolene suture (Ethicon, Norderstedt, Germany), the anterior chamber was entered with the needles through the paracentesis, the temporal inferior haptic was pierced with both needles, and the needles were extended under the scleral flap using the guided-needle technique.

The threads were tightened to gain a central final positioning of the mini telescope IOL and knotted.

On the first postoperative day (Fig. 1B), a centered Mini Telescope lens was seen, and the eye was normotensive at 14 mmHg. There was a progressive anterior chamber flare during the day, which completely resolved over the course of the next 4 days. An ultrasound examination showed an attached retina. Ten days after surgery the telescope was perfectly centered (Fig. 1C).

To the best of our knowledge, this is the first report of scleral fixation of a SING IMT™. The surgery showed that suture fixation is possible even without eyelets and may be a good solution in case of problems with intracapsular placement.
